# Initiating Aha moments when implementing person-centered care in nursing homes: a multi-arm, pre-post intervention

**DOI:** 10.1186/s12877-019-1121-3

**Published:** 2019-04-23

**Authors:** Laci J. Cornelison, Linda Hermer, Maggie L. Syme, Gayle Doll

**Affiliations:** 1 Center on Aging, Kansas State University, 253 Justin Hall, 1324 Lovers Lane, Manhattan, KS 66506 USA; 2 LeadingAge Center for Applied Research, 2519 Connecticut Ave. NW, Washington, DC 20008 USA

**Keywords:** Long-term care, Skilled nursing facilities, Culture change, Resident-centered care, Organizational change, Incentives, Pay-for-performance, Resistance to change, Barriers

## Abstract

**Background:**

Comprehensive adoption of culture change via person-centered care (PCC) practices in nursing homes has been slow. Change such as this, requires transformation of organizational culture, frequently generating resistance and slow moving change. This study examined how nursing homes perceive their adoption of PCC practices across seven domains and how these perceptions change in response to an educational intervention embedded in a statewide program, Promoting Excellent Alternatives in Kansas nursing homes (PEAK 2.0). Given perception is an important feature of the change process, it was hypothesized that pre-adopters engaging in PEAK 2.0’s initial Foundation year (level 0) would have *lower* perceived PCC adoption *following* a year of education and exposure to PCC, whereas adopters (PEAK 2.0 level 1–5 homes) would have higher perceived PCC adoption following a year of participation in their respective level in the program.

**Methods:**

A multi-arm, pre/post intervention study was conducted during the 2014 and 2015 years of the PEAK 2.0 program comparing pre-PCC adopters to adopters. Outcomes were self-ratings of perceived PCC implementation across seven domains of PCC at the beginning and end of the 2014–15 program year, after pre-adopters had received PCC education and adopters had implemented a year of PCC. Paired t-tests and mixed repeated-measures linear models, controlling for potential confounders, were employed to test the study hypotheses.

**Results:**

Across all seven domains of PCC, pre-adopters rated their PCC implementation as significantly higher on pre-test (2014) than on post-test (2015). In contrast, adopters rated their PCC achievement as higher on post-test (2015) than on pre-test (2014).

**Conclusions:**

Pre-adopters’ lower score following a year of education and exposure to PCC may reflect a shift in perceptions of PCC as a concept or a deeper conceptualization of PCC. Since perceptions or assumptions can serve as a source of resistance to change, redefinition or “unlearning” to make way for more accurate definitions of PCC could aid in reducing organizational resistance to implementation of PCC and thus improve the rate of adoption.

**Electronic supplementary material:**

The online version of this article (10.1186/s12877-019-1121-3) contains supplementary material, which is available to authorized users.

## Background

Since OBRA ‘87, nursing home reform has been a prominent national issue largely due to egregious cases of abuse, neglect and exploitation [1]. Early reform centrally focused on improving clinical quality in nursing homes, overshadowing quality of life. In the late 1990s, a new type of reform, culture change, entered into nursing home language, calling for improved quality of life and a shift away from institutional, medically-focused care [2]. Koren [3] and Harris, Poulsen and Vlangas [4], suggest that culture change includes; a) individualizing care, b) creating home-like environments, c) promoting close relationships between staff residents, families and communities, d) empowering staff to respond to resident needs and work collaboratively with management to make decisions about care and e) continuous quality improvement. Utilizing this framework, the Kansas Department of Aging and Disability Services (KDADS) developed the PEAK (Promoting Excellent Alternatives in Kansas Nursing Homes) program to encourage Kansas homes to adopt innovative practices specifically focused on promoting culture change. In 2011, KDADS released PEAK 2.0, which outlined a structured definition operationalizing culture change, the broad term, into specific person-centered (PCC) practices, and embedded it in a Medicaid pay-for-performance program [5].

KDADS’ self-proclaimed motivation for shifting to a pay-for performance program stemmed from low engagement of homes in adopting PCC practices and falling short of achieving culture change [5]. This is congruent with culture change adoption rates nationally. According to Miller, et al. [6], 85% of director-of-nursing staff interviewed reported at least partial involvement in culture change, leaving only 15% of respondents reporting little to no involvement. This appears promising on the surface; however, the data also indicated that only 13% of the respondents reported that culture change had “completely changed the way they care for residents” in “all areas of the nursing home” [6]. This is an increase from a previous study that noted only 5% of nursing homes reported that they had “completely changed the way they care for residents” [3]. However, it is widely acknowledged that culture change is meant to be comprehensive in nature rather than limited to individual components or practices [7]. A recent study has shown that the intended benefits to residents’ satisfaction with quality of life, as well as improvements to residents’ health, primarily accrue after comprehensive rather than partial adoption of culture change via PCC practices [8]. Thus, comprehensive adoption of culture change is the ultimate goal of the movement, with current research suggesting that 87% of nursing homes have not met this mark [6].

Why might comprehensive implementation of culture change in US nursing homes be limited, given it has been widely acknowledged as the answer to poor traditional care? One potential reason is that changing the culture is difficult, and requires deep organizational change. In the context of organizational theory, deep change is classified as a revolutionary change or “a major overhaul of the organization resulting in a modified or entirely new mission, a change in strategy, leadership and culture” [9] (pg.1). In contrast, most organization-level change is evolutionary or involves small continuous adjustments, while revolutionary change is a more monumental achievement. The thorough adoption of culture change in nursing homes mirrors revolutionary change in other sectors, requiring a complete change in the organization’s essential framework and affecting even the most basic capabilities. This type of change is manifest in everything from how the organizations’ employees interact with one another to how it fares in the marketplace.

As Burke [9] noted, revolutionary change involves changing culture, which is a widely discussed and studied component of organization change [10]. Changing culture is difficult due to the human forces that either facilitate or prevent transformation; for these reasons revolutionizing culture has been dubbed the “change monster” [11]. As one expert suggests, “Those who understand the challenge of culture change recognize the enormity of this task because it involves the creation of shared systems of meaning that are accepted, internalized, and acted on at every level of the organization”[12] (pg.143), and often resisted at every step [10–12]. Nursing homes are especially challenging because of their traditionally rigid worker hierarchies and strict regulations, as well as daily performance of many repetitive tasks, preponderance of low-skill and low-wage workers, and narrow operating margins [13].

### Organizational change

Organizational change theory is well canvassed in text and trade books; however, there are few empirical studies on the actual change process. Organizational change theories and models emphasize the dynamic nature of the change process [14]. For example, Lewin’s [15] classic three-stage model of change presents a three-step evolution; unfreezing, transition, and refreezing. In the unfreezing stage, organizations challenge the status quo and establish a need to depart from the existing equilibrium. The transition stage involves implementation or changing the mental structure by cognitive restructuring, semantic redefinition, and new standards of judgment. In the last step, refreezing organizations sustain changes and deeply integrate new values, traditions, and practices [16]. The transition stage is a particularly turbulent time for organizations as it is associated with disequilibrium, breaking down of old patterns and habits, experimenting with new ways, and developing what will be a new sense of homeostasis [17].

How and why do organizations enter the process of change? Journalist Malcolm Gladwell [18] (pg. 257) argues, for innovations to reach a tipping point or to become the norm it requires that “we reframe the way we think about the world”. Akgun, et al. [16], describe this reframing as a key function of organizational change, labeling it “unlearning,” which is a continuous process embedded in the organizational change process. “Unlearning involves the combination of the changes in beliefs and routines and these two components of unlearning must exist in tandem for unlearning to occur effectively” [16] (pg. 801). Perceptions or beliefs coupled with changes in routines are synergistic and happen dynamically, even catalyzing the change process [16], demonstrating not only the importance of changing practices which is often emphasized, but also the value of perceptions during change. What can initiate a shift in the way nursing homes’ perceive their work? In other words, what is needed for homes to have “Aha!” moments? Given the slow rate of culture change via person-centered practices in nursing homes: How can nursing homes be initiated or “unfrozen,” allowing for a transition to further change processes?

### Study rationale and purpose

While the challenges nursing homes face in initiating culture change are evident, successful mechanisms to advance change on a wide scale are scarce. The Kansas PEAK 2.0 program incents change in nursing homes on a wider scale, making this program a unique opportunity to evaluate a systemic approach to influence increased adoption of culture change via PCC on a larger scale. PEAK 2.0 is a Medicaid pay-for-performance program designed to incent person-centered care (PCC) practices. A broad stakeholder group assisted the Kansas Department of Aging and Disability Services (KDADS) in developing a standardized set of criteria for PCC practices, which represents an operationalized definition of the broader culture change concept. From here forward, the term PCC will be used rather than culture change, for specificity.

**Fig. 1:**
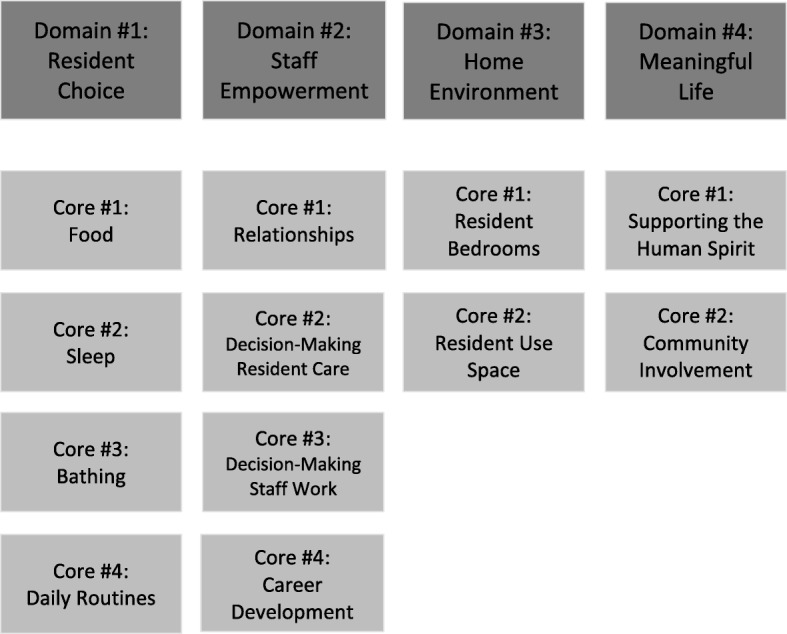
PEAK 2.0 domains and core areas

**Fig. 2:**
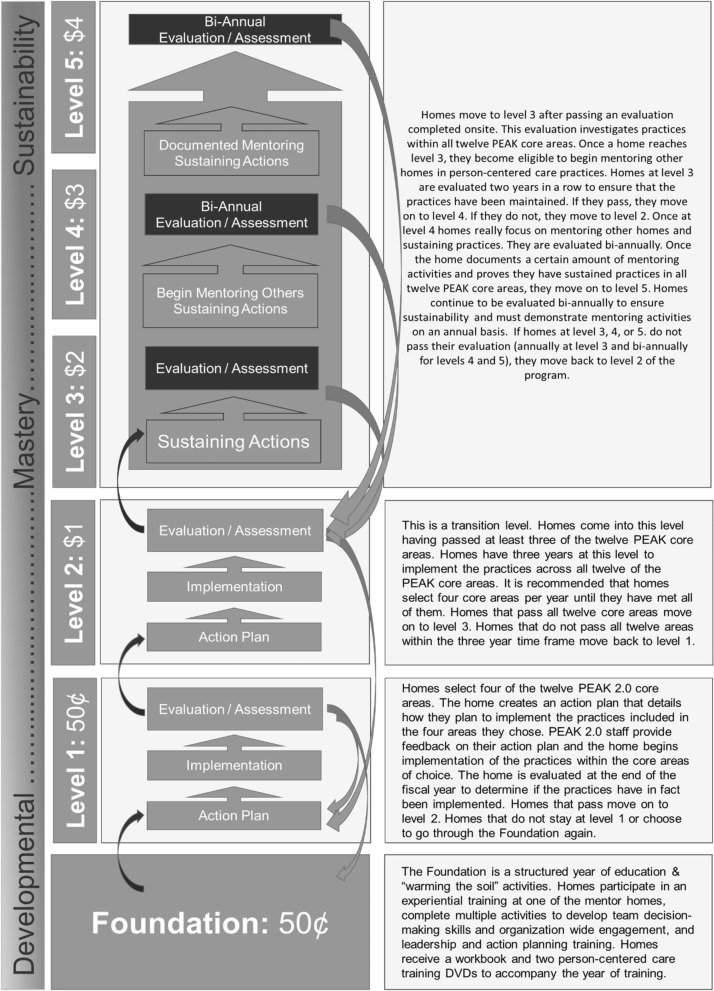
PEAK program overview: Levels and incentives. All incentive dollar amounts noted above indicate a per Medicaid resident, per day rate [31]

The PEAK 2.0 program is voluntary and was designed to financially reward both achievement and implementation of PCC adoption, which is outlined via the shared, standardized set of PCC criteria that includes 12 core concentration areas (Fig. 1). Homes that adopt increasing PCC practices can progress through six levels with corresponding, escalating financial incentives as a home moves from novice to mastery of PCC practices (Fig. 2). Novice homes start by completing a year of education and experiences designed to develop organizational readiness for change. Homes then begin implementing PCC practices, four core areas at a time, until they have implemented all 12-core areas of PCC. Then, homes work on sustaining practices and mentoring other homes in earlier stages of change. The 12 core areas of PCC practice are organized under four main program domains: a) resident choice, b) staff empowerment, c) home environment, and d) meaningful life. See Fig. 1 for a full listing of the domains and core areas.

Gaps in understanding of PCC were noted early in the program (2012) through review of participating homes’ action plans, which are detailed plans of how the home plans to implement the selected core areas. For example, some action plans detailed changes in practices that, if implemented, would not move them any closer to meeting the PCC criteria than doing nothing at all. Homes in this group often were often unaware they had a gap in understanding and in many cases perceived they were actively practicing PCC. As a result, the Foundation level was created in 2013 and includes education and exposure to PCC practices as well as guided team engagement and training in leadership skills. The Foundation level participants inspired questions about how the Foundation year affects homes’ perceptions of PCC and subsequent practices. Did their perceptions of practice change? If so, in what way(s)?

This study examines how nursing homes in the PEAK 2.0 program perceive their adoption of PCC practices, and how those perceptions may change across a year of education and experience, depending on their level of PCC practice adoption (pre-adoption vs. adoption). The Foundation level year will be targeted to determine how this group’s perceptions change in response to a yearlong educational intervention as compared to other program levels. Specifically, we hypothesized that, after accounting for key covariates: a) nursing homes categorized as pre-adopters of PCC practices (PEAK 2.0 level 0) engaging in the Foundation level year would have significantly lower perceived PCC adoption following a year of education and exposure to PCC, whereas b) nursing homes categorized as adopters of PCC practices (PEAK 2.0 levels 1 to 5) would have significantly higher perceived PCC adoption following a year of participation in their respective program level.

## Methods

### Participants

The sample consists of nursing home and staff-level data, and was drawn from homes participating in PEAK 2.0 from 2014 to 2015 that took the Kansas Culture Change Instrument (KCCI) in both of these years (*n* = 168). This represents 48% of the homes in Kansas. It has been shown that homes joining the program those 2 years were similar to homes not participating in the program on all studied demographic characteristics such as profit status and number of health deficiencies [19]. These are factors that had previously been shown to distinguish PCC adopters from non-adopters [20, 21]; however, the current sample’s demographics resembled those of nursing homes in the state as a whole [19]. In the sample of participants, homes are spread across various levels of the PEAK 2.0 program depending on their degree of PCC adoption, according to the KDADS PEAK 2.0 Criteria. In the analyses, homes were labeled by their level of PCC adoption (1 to 6) based upon their level in the PEAK 2.0 program at the beginning of the study (2014). For this study, the primary group of interest (Group 1: pre-adopters at PEAK level 0) consisted of Foundation-level homes that completed the KCCI prior to their Foundation level year of education and training (2014), took the KCCI again following their completion of the Foundation level (2015), and successfully passed to level 1 for the next year. Homes starting the Foundation level in 2014 but failing to complete it (*N* = 4) were omitted from the sample because they did not experience the intervention of education and training or fit in any of the other studied categories. The other groups of interest (Groups 2 to 6: adopters at PEAK levels 1 to 5), consisted of homes active in higher PCC levels of the program and together served as a comparison group in the analyses.

### Measures

#### The Kansas culture change instrument (KCCI)

All homes enrolled in the program self-evaluate their perceived adoption of PCC practices through the Kansas Culture Change Instrument (KCCI) annually. The KCCI is similar to the more familiar measurement tool Artifacts of Culture Change. The KCCI was utilized because it was specially designed for Kansas by a grant funded by the then Kansas Department on Aging before the Artifacts of Culture Change was widely released. The KCCI is a 68-item survey that measures self-reported perceptions of culture change implementation across seven dimensions: a) resident care b) nursing home environment, c) relationships, d) staff empowerment, e) nursing home leadership, f) shared values, and g) quality improvement [22] (p7). Homes rate their responses to survey statements on a four point Likert Scale (1- never; 2- sometimes, 3-often; 4- always). The KCCI dimensions have considerable overlap with the PEAK 2.0 domains, with each dimension often mapping onto more than one of the four PEAK 2.0 domains (refer to Additional file 1: Figure S3 showing dimension to domain connections). The exception is the KCCI dimension, related to continuous quality improvement, which is not represented in PEAK 2.0 domains. In each of the above named dimensions, participants respond to items/statements on a Likert scale from 1 to 4, with 1 being “never” and 4 being “always” [33]. Reliability studies showed Cronbach alpha scores ranging from .75 to .94 across the seven subscales [22] (p10). Three separate validity tests demonstrated adequate validity was achieved with the tool [22]. One original item in the nursing home leadership section was omitted (“Nursing home leaders ignore ideas from staff.”) due to reverse scoring issues. Homes in the PEAK 2.0 program were asked to have six team members fill out the survey that was administered online at PEAK 2.0 enrollment time. Of the team members filling out the survey, at least two were required to be direct care workers (e.g., certified nurse’s aides, housekeepers, dietary aides, and certified medication aides) and one was required to be either the administrator or the director of nursing. The other three participants could be anyone of the home’s choice. For most analyses, to represent home-level data, a single total score was computed by averaging six individual staff scores together for each of the seven survey domains noted above. For analysis of management verses direct care staff scores, the two direct care staff surveys were averaged together for a direct care staff score and the others were then averaged together for the management score. See Additional file 2 for the full survey.

### Demographic and other home-level data

To investigate direct care staff versus management scores, position title was collected. Additional nursing home baseline characteristics from 2014 and 2015 were obtained from Kansas Medicaid cost reporting data, which included staffing hours (combined aide, LPN, and RN hours-per-resident day), combined aide, LPN and RN turnover levels, resident acuity levels, and the percentage of Medicaid-funded residents.

### Procedures

All participants in the study completed the KCCI at the beginning of the PEAK 2.02014 program year. As part of the typical Foundation year, pre-adopters participated in structured education on PCC practices, visited a home providing comprehensive PCC practice, met virtually with experts on a regular basis, completed various team-building activities, and received training on leadership and action planning [23]. Activities across Groups 2–6 (adopters) were more independent in nature and focused on implementation of and/or sustaining PCC practices. At the culmination of the year, the PEAK 2.0 team provided feedback to and evaluation for both pre-adopters and adopters. Additionally, all participants again completed the KCCI.

### Statistical analyses

All data analyses were conducted using Stata v.12 IC (Statacorp LLC, College Station, TX). To determine whether the six groups differed on any baseline characteristic, one-way analyses of variance (ANOVAs) were conducted. These analyses were followed by Pearson correlation tests between continuous covariates and the seven continuous KCCI outcomes to help identify potential additional predictor variables. Covariates with significant associations with Group (pre-adopters vs. adopters), any of the outcome measures (KCCI dimensions), or both were included in the multivariate analyses. Descriptive statistics were computed for pre-adopters’ ratings of perceived PCC achievement in each of the seven KCCI dimensions by Year (2014 or 2015), and the two ratings were compared with paired t-tests. Primary analyses of pre-adopters’ results were seven multivariate, mixed repeated-measures ANOVAs with Year (2014 and 2015) as a repeated predictor variable, Group (pre-adopter vs. adopter) as a between-subjects predictor variable, and four covariates that were identified as potential confounders or predictors (i.e., staffing hours, turnover levels, acuity levels, and percentage of Medicaid residents). The dependent variables were each of the seven KCCI dimension scores.

Finally, pre-adopters were compared to adopters in their perceived PCC practice ratings by staff role. Multivariate, mixed repeated-measures ANOVAs were then conducted for each KCCI dimension score as dependent variables, with the repeated factor Year (2014 or 2015), the predictor variable Group (pre-adopters vs. adopters), Role (direct care worker or management-level staff member), and the four covariates identified in the preliminary analyses.

## Results

Table 1 presents the study’s six groups, with descriptive statistics for the groups’ baseline characteristics. All four covariates differed significantly by group, and in addition, some of them correlated with one or more of the KCCI outcomes.

**Table 1:** Groups 1 to 6 (1: Pre-adopters/2–6: Adopters) Descriptives and Univariate Comparisons

Group	Facility Count	2014 PEAK 2.0 Level	2015 PEAK 2.0 Level	Mean Staffing (SD)***	Mean % Turnover (SD)*	Mean Acuity (SD)**	Mean % Medicaid(SD)*
1	82	Foundation (0)	1	4.2 (1.0)	59.1 (33.6)	0.99 (0.01)	53.9 (17.4)
2	17	1	1	4.5 (0.74)	60.2 (26.5)	0.98 (0.07)	61.8 (11.2)
3	10	1	2	4.6 (0.51)	59.0 (26.0)	1.05 (0.90)	50.4 (20.9)
4	47	2	2	4.4 (1.0)	52.5 (23.9)	0.98 (1.0)	55.9 (19.0)
5	3	2	3–5	4.0 (0.3)	72.3 (33.7)	1.02 (0.2)	54.5 (4.1)
6	9	3–5	3–5	5.1 (0.81)	46.6 (8.6)	0.96 (0.10)	51.1 (22.5)

*Note.* Asterisks denote factors that differed by Group in univariate analyses: *p < 0.05, **p < 0.01, ***p < 0.005

Regarding the paired t-test results with pre-adopters KCCI scores, for all seven KCCI dimensions rated, the mean 2014 scores were significantly higher than the mean 2015 scores (Table 2). Multivariate analyses also revealed a significant effect of Year for all seven KCCI dimensions analyzed: Resident Choice (*F*(1,76) = 7.82, *p* = 0.0065), Nursing Home Environment (*F*(1,76) = 15.87, *p* = 0.0002), Relationships (*F*(1,76) = 20.78, *p* < 0.00005), Staff Empowerment (*F*(1,76) = 25.84, *p* < 0.00005), Nursing Home Leadership (*F*(1,76) = 14.31, *p* = 0.0003), Shared Values (*F*(1,76) = 12.28, *p* = 0.0008) and Quality Improvement (*F*(1,76) = 13.46, *p* = 0.0005). No other results from these analyses were significant.

**Table 2:** Pre-Adopters Scores by KCCI Dimension and Year

KCCI Dimension	Year of PEAK 2.0 (M, SEM)		
	2014 (*n* = 82)	2015 (*n* = 82)	t-values
Resident Choice	3.30 (0.042)	3.16 (0.041)	2.8514**
Nursing Home Environment	2.97 (0.034)	2.83 (0.042)	3.9114****
Relationships	3.16 (0.034)	3.01 (0.034)	4.4785****
Staff Empowerment	2.67 (0.042)	2.46 (0.045)	4.8952****
Nursing Home Leadership	3.04 (0.041)	2.85 (0.041)	3.8196****
Shared Values	3.43 (0.037)	3.28 (0.037)	3.4761****
Quality Improvement	2.90 (0.038)	2.76 (0.034)	3.6629****

*Note.* Results of the paired t-tests comparing each home’s ratings in the 2 years indicated by: **p < 0.01, ****p < 0.0005

Finally, pre-adopters were compared to adopters (Table 3) in each of the seven KCCI dimensions, and by staff role (Table 4). For the first KCCI dimension, Resident Choice, there was a significant effect of Group (pre-adopters vs. adopters) (*F*(1,643) = 96.24, *p* = 0.00005), with pre-adopters’ ratings being lower than those of adopters, reflecting their relative lack of achievement at implementing PCC practices. Additionally, there was a Group by Year interaction (*F*(1,643) = 14.19, *p* = 0.0002), with pre-adopters rating their perceived PCC achievement in this dimension higher in 2014 than in 2015, whereas adopters did the opposite, rating their perceived achievement in this dimension higher in 2015 than 2014. There was also an effect of Role (Table 4; *F*(1,643) = 4.87, *p* = 0.0277), with aides providing higher ratings than management (across Groups and other factors). None of the other variables were significant. For the second KCCI dimension, Nursing Home Environment, the findings were relatively similar. Again there was an effect of Group, with pre-adopter’s ratings overall lower than those for adopters (Table 3; *F*(1,643) = 71.34, *p* < 0.00005). Again, pre-adopters rated this domain higher in 2014 than 2015, opposite the pattern of adopters, as reflected in the significant Group by Year interaction (*F*(1,643) = 11.86, *p* = 0.006). In this case, there was no effect of Role, nor of any other variables in the model. For the third KCCI dimension, Relationships, again pre-adopter’s scores were lower than those of adopters (*F*(1,643) = 40.97, *p* < 0.00005), and again pre-adopters rated their perceived Relationship achievement as greater before their Foundation year than afterward, in contrast to adopters (*F*(1,643) = 12.98, *p* = 0.0003) who rated their perceived Relationships achievement as higher on post-testing (2015) than on pre-test (2014). Additionally, aides and other direct care workers rated this outcome as higher than management-level staff (Role: *F*(1.643) = 9.46, *p* = 0.0022). Finally, there was a modest effect of Turnover Percentage, with staff at facilities with higher turnover tending to rate their achievement along the Relationships dimension as lower (*F*(1,643) = 4.32, *p* = 0.0382). For the fourth KCCI dimension, Staff Empowerment, again there was an effect of Group (*F*(1,643) = 93.87, *p* = 0.00005) and a significant Group by Year interaction (*F*(1,643) = 13.78, *p* = 0.0002), with the same patterns as above. There was also an effect as before with Role (*F*(1,643) = 7.37, *p* = 0.0068), but no other significant associations. For the fifth KCCI dimension, Nursing Home Leadership, again there was an effect of Group (*F*(1,643) = 31.65, *p* < 0.00005) and a Group by Year interaction (*F*(1,643) = 11.96, *p* = 0.0006), both with the same patterns as above. Additionally, there was an effect of Role, again with direct care workers rating achievement as higher (*F*(1,643) = 26.90, *p* < 0.00005), and no other significant effects. For the KCCI dimension of Shared Values, the patterns were as with Nursing Home Leadership: a significant effect of Group (*F*(1,643) = 30.21, *p* < 0.00005), a significant Group x Year interaction (*F*(1,643) = 10.79, *p* = 0.0011), a significant effect of Role (*F*(1,643) = 8.64, *p* = 0.0034), and no other significant results. Finally, for the KCCI dimension of Quality Improvement, the pattern of results differed slightly. As before, there was a main effect of Group (*F*(1,643) = 32.13, *p* < 0.00005), with adopters rating their perceived Quality Improvement achievement in this domain higher than pre-adopters. However, there was also a significant effect of Year, with an overall preponderance of higher scores in 2015 than 2014 (*F*(1,643) = 5.58, *p* = 0.0144). Still, there was a marginally significant interaction between Group and Year, with pre-adopters tending to rate 2014 more highly than 2015, opposite the pattern of adopters (*F*(1,643) = 3.04, *p* = 0.0818). Finally, there was an effect of Role as with several of the prior dimensions: Aides rated their homes’ achievement in this area more highly than did management (*F*(1,643) = 22.20, *p* < 0.00005).

**Table 3:** Pre-Adopters vs. Adopters Scores by KCCI Dimension

KCCI Dimension	Year of PEAK 2.0 (M, SEM)	
	2014	2015
Pre-Adopters	**(*n*** **= 82)**	**(*n*** **= 82)**
Resident Choice***	3.30 (0.042)	3.16 (0.041)
Nursing Home Environment**	2.97 (0.034)	2.83 (0.042)
Relationships***	3.16 (0.034)	3.01 (0.034)
Staff Empowerment***	2.67 (0.042)	2.46 (0.045)
Nursing Home Leadership***	3.04 (0.041)	2.85 (0.041)
Shared Values***	3.43 (0.037)	3.28 (0.037)
Quality Improvement+	2.90 (0.038)	2.76 (0.034)
Adopters	**(*n*** **= 86)**	**(*n*** **= 86)**
Resident Choice	3.53 (0.041)	3.62 (0.028)
Nursing Home Environment	3.18 (0.046)	3.23 (0.038)
Relationships	3.29 (0.039)	3.32 (0.035)
Staff Empowerment	2.93 (0.057)	3.00 (0.048)
Nursing Home Leadership	3.15 (0.052)	3.20 (0.040)
Shared Values	3.53 (0.042)	3.58 (0.02)
Quality Improvement	3.05 (0.047)	3.02 (0.037)

Bold entries are for emphasis

*Note. M* Mean, *SEM* standard errors of the mean. Mean scores are averaged across management and direct care worker respondents. Asterisks indicate the significance of the test for an interaction between Pre-Adopters/Adopters and Year of PEAK 2.0: +*p* < 0.10, **p < 0.01, ***p < 0.005

-

**Table 4:** Direct Care Workers vs. Management Staff KCCI Scores by Dimension

	Role of Rater (M, SEM)	
	Direct Care	Management
KCCI Domain	(*n* = 344)	(*n* = 323)
Resident Choice*	3.43 (0.022)	3.36 (0.029)
Nursing Home Environment	3.06 (0.022)	3.03 (0.029)
Relationships***	3.22 (0.019)	3.12 (0.017)
Staff Empowerment**	2.80 (0.027)	2.69 (0.036)
Nursing Home Leadership****	3.12 (0.023)	2.91 (0.035)
Shared Values***	3.58 (0.020)	3.38 (0.031)
Quality Improvement****	2.97 (0.021)	2.81 (0.029)

*Note. M* Mean, *SEM* Standard Errors of the Means. Mean scores averaged across management and direct care worker respondents. Asterisks indicate the significance of the test for an effect of Role in the multivariate analyses: *p < 0.05, **p < 0.01, ***p < 0.005, ****p < 0.0001

## Discussion

This study examined changes in perceived PCC practice implementation through comparing staff perceptions of PCC practices before and after exposure to education and experience with PCC practices. It was hypothesized that pre-adopters would score lower on the KCCI after their year of education and training once individuals obtained more information about PCC and exposure to PCC in operation, and that the opposite pattern would be observed in the pre/post KCCI scores of adopters. Results showed that pre-adopter’s 2014 mean scores were higher than the mean scores for this group in 2015, as hypothesized. This pattern was present in all PCC dimensions of the KCCI survey, and persisted after controlling for several confounding nursing home variables. Pre-adopters entered the Foundation level with little background or operational practices in PCC, and then experienced a year of structured education and exposure to PCC practices. It is likely that the difference in mean KCCI scores is not simply a reflection of a true decrease in PCC practices in the pre-adopting homes, but rather, it represents a change in how participants define or conceptualize PCC practices, and culture change as a concept. Thus, the participants’ 2015 lower score values may reflect an enhanced understanding and awareness of true PCC practices, resulting in an adjusted assessment of their home’s performance in providing PCC after further education and exposure to a shared construct of PCC practices.

This is consistent with organizational change literature, which highlights the “unlearning” and “cognitive redefinition” associated with Lewin’s unfreezing step in the change process [15]. To illustrate, nursing homes at the pre-adopter level may be frozen in their operationalization of PCC practices, and unaware of their own frozen progress toward culture change. In other words, they do not know what they do not know about PCC practices, resulting in inflated pre-test KCCI scores. Schein [10] asserts that after some form of unfreezing, people and organizations become ready to learn, which makes way for cognitive redefinition. This is a three part process including; 1. Semantic redefinition: learning that words can mean something different than assumed, 2. Cognitive broadening: learning that given concepts can be much more broadly interpreted than assumed, and 3. New standards of judgment or evaluation: the realization that anchors we used for judgment and comparison are not absolute leading to judgment shift.

These findings provide some evidence that the Foundation year, which embodies education and shared experiences, might ignite the unfreezing process in participating organizations, which is postulated as key to the next steps in the change process [15]. Akgun and colleagues [16] assert that unlearning can catalyze the organizational learning process and make way for a more dynamic learning process. Colloquially, people undergoing a change process often refer to having an “aha” moment (personal observation). In this study, the pre-adopters may achieve these “aha” moments as a part of their Foundation activities, as they realize what they did not know (i.e., unfreezing) and begin to redefine PCC practices and adjust their perceived performance in the KCCI dimensions, resulting in lowered scores across the program year.

Another finding of interest in this study was the importance of staff role in perception of PCC practices. Across most PCC practices, nurse aids reported significantly higher PCC implementation than management across all other factors. This may be due to direct care staff having closer contact with day-to-day practices and thus are better able to perceive PCC practices. Higher scores may also be due to the pride direct care staff feel toward their difficult and important work, which is reflected in higher ratings. In contrast, the Nursing Home Environment dimension did not show significant differences. This might be due to the fact the environment is more tangible resulting in fewer differences among the roles within the home. Difference between management and front line worker perspectives is consistent with other studies [24–26]; however, literature is mixed in which group rates performance higher than the other. Difference between manager and frontline worker perspectives should be explored further, and this finding highlights the importance of garnering multiple perspectives to develop a more accurate representation of overall organizational performance.

### Limitations

The following limitations should be considered when interpreting the results of this study. First, the measurement of KCCI scores pre and posttest represent perceptions of PCC practice implementation, and serve as a proxy measurement in this study, not fully aligning with the PEAK 2.0 Criteria. However, as evidence by Fig. X, there is considerable overlap in the KCCI tool and the Criteria making it a plausible proxy for measure perceptions of culture change implementation. Secondly, this study employed a quasi-experimental design using pre-existing groups, which limits the ability to draw causal inferences. This limitation is, unfortunately, endemic to field work, and is attenuated by the consistent findings across PCC dimensions and the ability to account for several potential confounding factors in the analyses.

Finally, this study utilized a convenience sample of Kansas nursing homes that all chose to enroll in the PEAK 2.0 program, which creates selection bias and limits the generalizability of the results. First, those that self-selected to enroll in the PEAK 2.0 program may be significantly different from Kansas’ nursing homes that chose not to enroll in ways that would affect the results. However, a recent study on the differences between PEAK 2.0 enrolled and non-enrolled nursing homes in Kansas—that included the current study’s sample and time period (2014 to 2015)—found enrollers to be similar to non-enrollers across several important characteristics (e.g., profit status, CCRC affiliation, urbanicity, percentage of Medicaid and Medicare residents [29]. Another potential limit to generalizability is that nursing homes in Kansas may be unique to other states because of any state regulatory differences specific to Kansas, financial incentive program promoting PCC practices, and limited diversity of the overall Kansas nursing home resident population as compared to other states (higher proportion of white, female residents). However, the consistency of the findings across PCC dimensions enable confidence in the conclusion that in order to enact deep change, nursing homes may need education and training (such as that of the Foundation year) to address perceptions of PCC when implementing it into practice.

### Implications

This study provides support for the occurrence of a change in perception of PCC practices among participants of the PEAK 2.0 program following participation in the Foundation level. Foundation level homes are novices to PCC provision. Within the Foundation year, homes do not undergo tangible changes to their home’s practices, but do undergo education and exposure related to PCC. The most significant finding in this study is that pre-adopters (Foundation level homes) rate themselves higher in multiple domains of PCC prior to participating in the Foundation activities than following participation in these activities. Since perceptions of PCC (i.e., how people conceptualize the concept) can serve as a source of resistance to change [27–29], altering peoples’ perceptions could aid in unfreezing nursing home organizations to move to the implementation (or transition step) of PCC practices. This realignment could be an indication of a more accurate conceptualization of PCC practices, and thus may aid the homes in truly implementing PCC practices through the shared conceptualization outlined in PEAK 2.0 criteria. The culture change movement intended for facilities to thoroughly adopt PCC practices, but thus far has resulted in homes being partial, not full adopters, as of 2014 [6]. The results of this study have implications for how the culture change movement could initiate awareness and implementation through education and experiential activities, potentially improving the rate of comprehensive adoption of PCC practices.

These findings also highlight the importance of education and training prior to change implementation. Organizational change literature tells us that knowing “what” or the overall direction for change is essential to success [9]. The significant change in perceptions of PCC practices before and after the initial Foundation year in PEAK 2.0 tell us that education and training likely impacted participants’ views of PCC, which has impact on their actions. Because several members from each organization (nursing home) collectively received the same type of education and training, there is a greater opportunity for cohesion in a purpose and vision for PCC, leading to a greater chance of unity and action going forward.

PEAK 2.0 is a statewide, voluntary reimbursement program to incent PCC, and other states desiring to promote culture change via PCC practices in nursing homes could look to this program as a model for actualizing PCC implementation, paying special attention to the Foundation level components. The results have similar implications for corporations, chains and individual operating homes that want to implement PCC practices. The primary message is that a shared definition of PCC practices and upfront education and exposure are important to understand current perceptions associated with PCC practices and encourage the unfreezing process of change.

### Practice and policy

On a micro level, organizational leaders such as CEOs, administrators, and directors of nursing, can use the findings from this study to aid in increasing awareness and accurate understanding of PCC practices vital to the culture change process. Though individual organizations outside of Kansas do not have access to the Foundation level of PEAK 2.0, the resources developed within the program are free and accessible online. The components incorporated within the Foundation level, which have demonstrated success, can be adapted by individual organizations, avoiding the need to purchase or recreate them.

On a macro level, statewide and national initiatives can use the findings of this study when designing policies or programs to promote culture change via comprehensive PCC practices. Such policies and programs should acknowledge the presence of potential misperceptions of PCC practices that may serve as barriers to change and address these barriers accordingly. Leaders in the culture change movement noted that one of the challenges of realizing success is that homes were attracted to the “low hanging fruit” [30] or partial, short-term, easier changes. Targeting “low hanging fruit” is, at least in part, a result of assumptions or misperceptions that prevent transition to comprehensive PCC practices, limiting the ability of homes to achieve deep organizational change. The findings of this research reveal ways for the culture change movement to overcome the “low hanging fruit” mentality and move to deep organizational change on a large scale.

## Conclusion

Across all seven dimensions of PCC assessed, pre-adopters rated their PCC implementation as significantly higher on pre-test (2014) than on post-test (2015), as hypothesized. In contrast, adopters rated their PCC achievement as higher on post-test (2015), after a subsequent year of PCC implementation, than on pre-test (2014). Pre-adopter’s lower score following a year of education and exposure to PCC, may be reflective of a change in how participants perceive or conceptualize PCC practices, rather than a decrease in PCC practices in the home. Since misperceptions and assumptions can serve as a barrier to change, altering staff perceptions could aid in unfreezing nursing home organizations, and move them into the process of change through initiating “aha” moments. Then, homes may move deeper into the implementation process of PCC practices, and thus improve the rate of comprehensive adoption. This study has implications for both individual homes implementing culture change as well as larger scale policy implementation.

## Additional files

Additional file 1: **Figure S3.** Cross-Reference of KCCI to PEAK 2.0 Domains. This is a figure created to show the significant overlap of the KCCI to the PEAK 2.0 Domains. (DOCX 50 kb)

Additional file 2: KCCI Survey. Kansas Culture Change Index Survey Instrument. The additional file is the survey that was used for the data collection in this study. (DOCX 31 kb)

## Abbreviations

ANOVAs: Analysis of Variance- a statistical technique to evaluate the difference among group means
KCCI: Kansas Culture Change Instrument- A survey tool to measure a nursing home’s level of adoption of culture change
KDADS: Kansas Department for Aging and Disability Services
KSU: Kansas State University
LPN: Licensed Practical Nurse
PCC: Person-Centered Care- a philosophy of care in nursing homes that changes the focus of caregiving from accomplishing tasks to emphasizing the person. As a result, the personal preferences of residents become as important as providing the services and supports they need. Person-centered care requires a shift in an organization’s values and beliefs about quality. Traditionally, high quality clinical care is seen as the pinnacle of a high quality nursing home. With person-centered care, high quality clinical care remains critically important, but quality of life is valued as equally important
PEAK 2.0: Promoting Excellent Alternatives in Kansas Nursing Homes- A pay-for-performance program to incent adoption of person-centered practices in nursing homes
RN: Registered Nurse
