# Supplementary material for: Initiating Aha moments when implementing person-centered care in nursing homes: a multi-arm, pre-post intervention
Source: BMC Geriatr. 2019 Apr 23;19:115. doi: 10.1186/s12877-019-1121-3 (PMC6480647; doi:10.1186/s12877-019-1121-3)
Supplement: Supplementary file 1 — Figure S3. Cross-Reference of KCCI to PEAK 2.0 Domains. This is a figure created to show the significant overlap of the KCCI to the PEAK 2.0 Domains. (DOCX 50 kb) [file 12877_2019_1121_MOESM1_ESM.docx]

Additional file 1: Figure SS3: KCCI Dimensions cross-referenced with the PEAK 2.0 Domains

PEAK 2.0 Domains

Domain #1:

Resident Choice

Domain #2: Staff Empowerment

Domain #3: Home Environment

Domain #4: Meaningful Life

KCCI 7 Dimensions

Quality Improvement

Shared Values

NH Leadership

Resident Care

Relationships

NH Environment

Staff Empowerment
