# Supplementary material for: Initiating Aha moments when implementing person-centered care in nursing homes: a multi-arm, pre-post intervention
Source: BMC Geriatr. 2019 Apr 23;19:115. doi: 10.1186/s12877-019-1121-3 (PMC6480647; doi:10.1186/s12877-019-1121-3)
Supplement: Supplementary file 2 — KCCI Survey. Kansas Culture Change Index Survey Instrument. The additional file is the survey that was used for the data collection in this study. (DOCX 31 kb) [file 12877_2019_1121_MOESM2_ESM.docx]

KCCI Survey

Instructions: "Culture change" is an effort to make a nursing home less like an institution and more like a home while maintaining quality of life for those who live and work there. Core Values include choice for residents, improving quality of care, staff empowerment and creating a homelike setting. Tell us about your nursing home. Please answer each question, as you believe it really is, not as you think it should be. For the following questions please answer with the response that best describes the way you feel about each question.

Q1: What nursing home are you currently employed with? If you are unable to find your home name below please contact the K-State Center on Aging at

<Drop down menu with all the homes in Kansas listed>

Q2 How long have you worked with (Home Name)?

- Less than 1 month
- 1-3 months
- 4-6 months
- 7-10 months
- 1-5 years
- 6-10 years
- 7-11 years
- 11-15 years
- 16-20 years
- More than 20 years

Q3 Do residents at (Home Name) live in small households or neighborhoods?

- Yes
- No

Q4 In what role do you spend most of your time at (Home Name)?

- Administrator
- Director of Nursing
- CNA / CMA
- Activities
- Maintenance
- Nursing
- Housekeeping
- Laundry
- Food Services
- Sales / Marketing
- Other ________________________________________________

Q5 What is your gender?

- Male
- Female

Q6 Please select the option that best describes your response.

|  | Never | Sometimes | Often | Always |
| --- | --- | --- | --- | --- |
| Residents choose when they eat each meal. |  |  |  |  |
| At mealtime, residents help themselves or tell staff what they want to eat. |  |  |  |  |
| Residents choose the time of day they bathe. |  |  |  |  |
| Residents choose the way they bathe (for example shower, bed bath, or bathtub). |  |  |  |  |
| Care plans are based on residents' request. |  |  |  |  |
| Residents can sleep late and still get breakfast. |  |  |  |  |
| Residents go to bed for the night at any time they want. |  |  |  |  |
| The nursing home has activities designed for residents with memory problems. |  |  |  |  |
| Residents who are able, dress themselves even if it takes a long time. |  |  |  |  |

Q7 Please select the option that best describes your response.

|  | Never | Sometimes | Often | Always |
| --- | --- | --- | --- | --- |
| Residents decorate their own rooms. |  |  |  |  |
| Residents can meet with visitors in a living room shared by a small group of residents. |  |  |  |  |
| Residents eat in a dining room shared by a small group of residents. |  |  |  |  |
| This nursing home has live indoor plants and flowers. |  |  |  |  |
| This nursing home has pets here. |  |  |  |  |
| Children from the community come to visit residents. |  |  |  |  |
| This nursing home looks and "feels" like home. |  |  |  |  |
| Spur of the moment activities happen here. |  |  |  |  |
| This nursing home displays residents' personal items, such as family photos, in common living areas outside their rooms. |  |  |  |  |
| Residents can get to outdoor spaces without staff help. |  |  |  |  |

Q8 Please select the option that best describes your response.

|  | Never | Sometimes | Often | Always |
| --- | --- | --- | --- | --- |
| Staff work with the same group of residents. |  |  |  |  |
| Families know who takes care of their loved ones. |  |  |  |  |
| The outside community is involved in nursing home activities. |  |  |  |  |
| Staff meet with family members to explain their role in their loved one's care. |  |  |  |  |
| Families visit their loved ones. |  |  |  |  |
| This nursing home has community volunteers. |  |  |  |  |
| Children from the community participate in programs with residents in the nursing home. |  |  |  |  |
| This nursing home takes time to remember residents who die. |  |  |  |  |
| Residents and staff are encouraged to talk about their feelings when a resident dies. |  |  |  |  |
| Residents choose to spend time with each other on their own. |  |  |  |  |

Q9 Please select the option that best describes your response.

|  | Never | Sometimes | Often | Always |
| --- | --- | --- | --- | --- |
| Direct care staff have input into resident care planning. |  |  |  |  |
| Certified aides take part in resident care plan meetings. |  |  |  |  |
| Direct care staff know when a resident's care plan has been changed. |  |  |  |  |
| Staff teams create their own work schedules. |  |  |  |  |
| Staff work together to cover shifts when someone can't come to work. |  |  |  |  |
| Staff are cross-trained to perform task outside of their assigned job duties. |  |  |  |  |
| This nursing home gives raises and other rewards to staff who receive extra training or education. |  |  |  |  |
| Direct care staff take part in quality improvement teams. |  |  |  |  |
| Staff are empowered to contact family directly when a resident has a personal need. |  |  |  |  |
| Staff grow as individuals here. |  |  |  |  |

Q10 Please select the option that best describes your response.

|  | Never | Sometimes | Often | Always |
| --- | --- | --- | --- | --- |
| Nursing home leaders value team members from all departments. |  |  |  |  |
| Decisions in the home are made by teams that involve direct care staff. |  |  |  |  |
| Nursing home leaders hire staff who really care not "just anyone". |  |  |  |  |
| Nursing home leaders try to improve working conditions. |  |  |  |  |
| Nursing home leaders ask questions with an open mind. |  |  |  |  |
| Nursing home leaders are available when staff need to talk. |  |  |  |  |
| Supervisors treat aides with respect. |  |  |  |  |
| Exit interviews are conducted when staff leave. |  |  |  |  |
| Changes in operations are made as a result of exit interview data. |  |  |  |  |

Q11 Nursing home leaders and staff share value and common goals related to:

|  | Never | Sometimes | Often | Always |
| --- | --- | --- | --- | --- |
| Homelike environment |  |  |  |  |
| Choice for residents |  |  |  |  |
| Respect for residents |  |  |  |  |
| Respect for co-workers |  |  |  |  |
| Decision making |  |  |  |  |
| Quality of life for residents |  |  |  |  |
| Quality of work life for staff |  |  |  |  |

Q12 Please select the option that best describes your response.

|  | Strongly Disagree | Disagree | Agree | Strongly Agree |
| --- | --- | --- | --- | --- |
| Staff turnover at this nursing home is low. |  |  |  |  |
| This nursing home evaluates our care and services to make improvements. |  |  |  |  |
| The data we collect helps identify problems with services. |  |  |  |  |
| This nursing home has a plan for lowering turnover. |  |  |  |  |
| This nursing home actively tried to keep employees working here. |  |  |  |  |
| Staff are updated about budget and cost changes. |  |  |  |  |
| Direct care staff, including aides, have input into the budget to care for their residents. |  |  |  |  |
| Staff ideas are used to reduce wasted time and effort. |  |  |  |  |
| The leadership team discusses staff turnover. |  |  |  |  |
| This nursing home has a plan to increase staff retention. |  |  |  |  |
| The leadership team uses MDS reports for quality improvement initiatives. |  |  |  |  |
| Direct care staff attend quality improvement meetings. |  |  |  |  |
